# Advice upon Medical Subjects

**Published:** 1869-10

**Authors:** 


### Advice Upon Medical Subjects.

BY THE EDITOR.

MUSTARD POULTICES.—Should be made with luke-warm water, rather than hot water. Its effect is destroyed by use of extreme heat.

---

HOW TO TAKE SALTS.—A favorite physic with many people is sulphate of magnesia (salts.) The bitterness of this medicine can be entirely disguised by dissolving it in strong coffee, instead of water. In fact, it is rather an agreeable drink than otherwise.

---

TO ALLAY A COUGH.—Take equal parts of honey, rum, and lemon juice. Heat it over a slow fire, and take a tablespoonful frequently during the day. We are constantly in the habit of prescribing this mixture in "severe colds," and find nothing equal to it in allaying irritation in the throat or lungs.

---

PAINFUL CORNS.—Take a piece of lemon peel, the size of the corn, to which the pulp of the lemon is attached, and bind it upon the corn every night, upon retiring. Two or three applications usually destroys the corn, rendering the parts soft and pliable. A cranberry bound on in the same manner, with the pulp next the corn, will give great relief.

---

BEAUTIFYING THE COMPLEXION.—A young lady of New Haven took arsenic for the purpose of rendering her complexion whiter, last week. She succeeded, and was placed in a box next day, looking beautifully white and calm. She was dressed in white muslin, with a wreath of beautiful white flowers upon her breast, and Amalaki John says she looked like a wax-doll. If any of our young lady friends want the recipe for the dose necessary to be taken, we can send it them upon application.

It is said the funeral of the young lady mentioned was largely attended.

---

CATARRH.—This is the season of the year when persons suffer most from catarrhal affections. Snuffing salt water through the nostrils into the mouth, two or three times per day, and taking salt baths three times a week, using much friction for the body, and changing the underclothing at every bath, will give great relief from the distressing symptoms.

The water snuffed into the nostrils, and used for the bath, should be luke-warm, with about a teaspoonful of salt to the quart of water.

Never use the advertised snuffs and "catarrh remedies," that we see puffed in almost every journal—they are worse than useless, and are often absolutely hurtful.

---

THE PERIOD FOR WOMEN TO MARRY.—M. A. Raciborski, a celebrated physician of Paris, says the only proper time for woman to marry is between the 20th and 24th years—and he's right. Our American girls are altogether too hasty in their marriages. If they would remain in the kitchen longer (or at all) and fit themselves for wives, by gaining a knowledge of household duties and adding more to their *physique*, they would make much better wives and mothers and rear far healthier children.

But the thought uppermost with girls now-a-days is, how can I catch a husband, and when? The mother generally assists the girl in her undertaking, and is generally only satisfied when her daughter has made a "good match," and the earlier the better. This is wrong, and such mothers and girls are responsible for two-thirds of the sickly, puny children we see upon our walks daily.

---

PIN WORMS.—In the last number of the BISTOURY we gave a plan for destroying those pests known as "pin worms." The process is simply to annoint the anus with lard or oil, twice a day, with a view of destroying the eggs of the animal, which are deposited on the outside of the sphincter. The animal is very short lived, so that by destroying their eggs in this manner, the tribe soon becomes extinct. We have seen several persons who have tried the plan suggested by us, and who report a complete cure of the difficulty. Others have written us upon the subject, thanking us for suggesting means for their relief, which had acted so promptly, when they had expended hundreds of dollars in seeking a remedy for the annoyance.

The fact is then established—*annointing the anus with oil will destroy pin worms!*

---

ABORTION DEPOPULATING THE AMERICAN PEOPLE.—We see it stated in the *Med. Archieves* that "if it were not for foreign immigration, and the births arising therefrom, the States of Massachusetts, New Hampshire, Connecticut, and Rhode Island would, in all probability, be depopulated in less than a century. Statistics show that where one child is born in New England, from four to five were born twenty-five years ago." We need not go to New England to see this discrepancy in the American population. Just visit any of the public schools of this or any other city, and you will see that the children of the Irish alone, will outnumber ours ten to one, in an equal number of families.

When mothers think less of fashionable calling and the yearly visitation of watering places, and pay more attention to household duties, we will have more American children and less criminal abortion.

---

ARTIFICIAL RESPIRATION.—It is always well to know how to proceed in cases of drowning of children, or in asphyxia of new-born infants. For the want of such information many helpless little ones have been allowed to die that might have been restored to life.

In drowning, apply your mouth to that of the child, hold its nostrils, and by exhausting the air in your lungs inflate the stomach of the child; then press upon its abdomen with a quick motion, when the water swallowed will be expelled. Repeat this manouvre two or three times, then lay the child upon its back and raise it upright to a sitting posture, when the weight of the vicera and liver will draw the muscles down and imitate inspiration. Then lay the child upon its back again, and make pressure upon the abdomen, when the diaphragm will be forced upward, imitating expiration. Continue this process for a half hour or more, unless natural breathing is sooner established, while the bystanders are applying friction and heat to the extremities.

In asphyxia of the new-born infant, lay the child upon its back, press gently upon its abdomen—then raise it to a sitting posture—again laying it upon its back, and so continuing this manouvre until you are rewarded with a cry from the infant. We have seen several infants restored in this manner that were supposed to be past recovery.

This is the most simple and effectual means of artificial respiration that we know of, and by giving it to our readers have answered several letters of inquiry upon the subject.

CHILDREN'S EARS.—Children often amuse themselves by thrusting peas, beans, pebbles, and other objects into their ears, and parents in attempting to remove these foreign bodies, are apt to injure the hearing apparatus. Probing or picking should never be resorted to, as the offending particle is invariably thrust further into the ear, and often against the drum, where it produces serious inflammation.

Procure a small glass or rubber syringe, and inject warm water carefully into the ear. The return flow of the water will generally bring the foreign particle with it. But if it should not, take your little one to a surgeon, who will remove it by means of a little syringe kept for the purpose.

We have seen many children, whose ears have been ruined by an awkward attempt at the removal of such particles—hence the warning.

---

LANCING CHILDREN'S GUMS.—We believe much unnecessary pain is caused our babies by the useless habit of lancing their gums, with a view of relieving pain from pressure of the tooth. This process is never necessary, unless the tooth presses firmly against the gum, causing an inflamed and swollen condition of the parts. We have seen physicians thrust a gum lancet into a baby's mouth, one year old, and cut the gums of its *double teeth.* Such a procedure is causing the infant unnecessary pain, besides absolutely doing it an injury; the scar from the cutting rendering the gum hard and gristly, through which the teeth must force their way, a year or so later. As a general thing, refuse to have your babies' gums lanced; it is usually an unnecessary performance.

---

BOWEL COMPLAINTS.—Look out for your children who are large enough to run about, climb fences, and pilfer fruit. It is as natural for a child to take fruit wherever they find it as it is for a duck to take to water.

When you find them (the children—not the ducks) complaining of belly-aches, and you have reason to suppose it is caused by a too free use of green fruit, don't give them a dose of laudanum to quiet them, but, if the symptoms are sufficiently alarming, give a child six years old, a half teaspoonful of mustard, mixed in a tumbler full of luke-warm water, and cause him to drink every particle of it. If he complains of feeling sick, give him a glass of warm water to "settle his stomach"—you will soon have the satisfaction of seeing him disgorge a peck—more or less—of the fruit upon which he feasted—a speedy cure will follow.

If, however, your little one should have what is termed a "bowel complaint"—too frequent discharges, accompanied with more or less pain and smarting; have the following medicine prepared, and give a child of six years, 15 drops in some sweetened water, every 3 hours, until the symptoms subside:

Take—
Tincture of Opium,
" Capsicum,
" Camphor, of each one ounce; mix, and give in doses as above described. This is a good remedy to keep constantly in the house, as it is useful in all manner of pains in the stomach and bowels.

---

TO PREVENT THE SPREAD OF TYPHOID FEVER.—This is very important, and the heads of families should be acquainted with the means of preventing the spreading of this terrible disease when it attacks a member of their family.

The poison, by means of which the typhoid fever spreads, is nearly entirely contained in the discharges from the bowels.

These discharges infect the air of the sick room; the bedclothing and linen of the patient; the privy or cesspool where such discharges are thrown; and it is not unfrequently the case that the poison finds its way into the well, infecting the drinking water, and so giving rise to the most deadly form of the poison.

Often, entire neighborhoods become infected from one patient, causing sad havoc among the inhabitants. A little care to carry out the following plan will invariably prevent the spread of the disease. This can be done by disinfecting the discharges as soon as they escape from the body. Procure one and a half pounds of "green copperas"—it is very cheap—dissolve it in a gallon of soft water—place a teacupful of this solution in the bed pan every time before it is used by the patient. This will render the discharges perfectly harmless. Should the bed clothing become soiled, rub chloride of lime upon the spots immediately. The clothing should be carefully inspected after every discharge, for this purpose. Sprinkle the chloride of lime under the bed, and have several saucers full standing about the room. Change the bed linen frequently, and place those removed into a pail of water containing a tablespoonful of the chloride of lime. The nurse in attendance upon the sick should not visit other families during the illness of those upon whom she is waiting, and upon the recovery of her patient, should thoroughly disinfect her clothing by soaking in the chloride of lime solution, and by exposing them to the air for two or three days. In case of death, the coffin should be sprinkled with the chloride of lime (under the corpse,) and the entire house thoroughly ventilated and sprinkled with the disinfectants.

Cesspools and privies should have large quantities of the chloride of lime thrown into them daily.

Where these directions are strictly carried out, the infection rarely spreads beyond the family or person first attacked. Where they are neglected, entire communities fall victims to the disease.

Let families observe these safeguards strictly this fall, and we will have much less of typhoid fever among us.

